# Four-year diploma male students’ experiences in a profession traditionally perceived as a female domain at a selected public college of nursing in Limpopo, South Africa

**DOI:** 10.4102/curationis.v41i1.1932

**Published:** 2018-10-04

**Authors:** Ntombizodwa P. Ndou, Salaminah S. Moloko-Phiri

**Affiliations:** 1 Department of Health, Limpopo College of Nursing, South Africa; 2 Department of Nursing Sciences, University of Pretoria, South Africa

## Abstract

**Background:**

Over the last 20 years, more men have been choosing to pursue a career in nursing. Despite this, men remain a minority in the nursing workforce around the world, including South Africa. Even though there is an increase in the number of male students entering the profession, male students remain a minority. Comparatively speaking, there is no balance between the number of female and male students taken in each intake. This is even reflected in the South African Nursing Council statistics. Nursing is traditionally perceived as a distinctly feminine career and the use of the terms ‘angel in a white dress, sister or nurse’ perpetuates this stereotype. This perception may deter some men from choosing a career in nursing and presents challenges for others who are currently in the profession.

**Objectives:**

To explore and describe 4-year diploma male students’ experiences in a profession traditionally perceived as a female domain.

**Method:**

This qualitative study used an explorative and descriptive design. The population comprised African male students in their third and fourth years of study who were registered for the 4-year diploma course at a selected public college of nursing in Limpopo, South Africa. Participants were purposively sampled. Five focus group discussions were conducted, and each comprised six to eight participants. The data were analysed using the Tesch’s open code method.

**Results:**

Two themes emerged during data analysis: discrimination in the clinical setting and lack of role models and mentors.

**Conclusion:**

Unless these challenging experiences are attended to, discrimination on the basis of gender in the clinical settings and lack of mentors may deter some men from choosing and remaining in the nursing profession. The selected public college of nursing and the nursing services need to work towards creating a welcoming environment to all students regardless of gender because some participants indicated that the clinical environment is sometimes not welcoming to them as men.

## Introduction

Nursing is perceived as a traditionally feminine profession, a stereotype that may be closely linked to Florence Nightingale’s approach to care. Additionally, Nightingale regarded women as carers and regarded it as a natural phenomenon for women to care for the sick; as a result, the role expectations of caring and being gentle are now more often aligned with women than with men (McWilliams, Schmidt & Bleich 2013; Brown & Crawford as cited by Ross 2017). Consequently, many men, because of these deeply entrenched gender expectations also consider nursing to be a female profession and are thus deterred from pursuing a career in nursing (Lou et al. 2007). Over the last 20 years, more men have chosen nursing as a profession, but they remain in the minority in most parts of the world including South Africa (South African Nursing Council [SANC] Statistics 2013). In the same vein, to demonstrate that male nurses are still in minority in South Africa, the South African Nursing Council Stats, 1/2017, confirm the numbers of nurses in Limpopo including those of male nurses as compared to those of female nurses as: female registered nurses, 10 567; male registered nurses, 1549; female enrolled nurses, 5833; and male enrolled nurses, 645; whilst female auxiliary nurses were 9300 and male auxiliary nurses were 825.

The few men in the nursing profession have an impact on male students who are currently enrolled. Male students lack male role models and mentors both at colleges and in clinical settings. As a result, male student nurses often feel isolated and frustrated during their training (Buthelezi et al. 2015; Stanley 2012). Men who enter the nursing profession often suffer much prejudice from both the public at large and from their female counterparts. Some male nurses have even been questioned about their sexuality after enrolling for nurse training (Achora 2016). Wang et al. (2010) reported that male students often experience social pressure, resulting in the perceived need to hide that they were nurses because they felt ashamed to be in the nursing profession. The use of distinctly feminine terms, for example ‘sister or matron’, has perpetuated a perception of a feminine profession where male nurses often felt left out including at the selected clinical learning environment where students are placed. These terms are used regularly to describe a woman of a certain role in the nursing profession. Similarly, in a study conducted in Cyprus, it is argued that it was an acceptable general perception that women nurses are the ones fitting and appropriate to provide care to women especially with procedures that are performed on the private parts (Kouta & Kaite 2011). This study explored and described 4-year diploma male students’ experiences in a profession traditionally perceived as a female domain at a selected public college of nursing in Limpopo, South Africa. The study was qualitatively explored.

## Research design and methods

A qualitative, exploratory, contextual and descriptive approach was employed (Polit & Beck 2013). A qualitative approach was chosen in order to explore and describe the experiences of male student nurses in a profession traditionally known as a female domain by collecting rich narrative data. An exploratory and descriptive approach was used to explore and describe the male students’ experiences. The study was contextual because the focus groups were conducted at the selected public college of nursing where the students were enrolled for a 4-year diploma programme.

### Population and sampling

The population consisted of African male student nurses registered for a 4-year diploma course according to South African Nursing Council Regulation, R425 of February 1985 as amended, at a specified college of nursing in Limpopo. Participants who would be able to give a relevant data were purposively sampled. Participants were included if they were men and in their third or fourth year of training. Male students in their third and fourth years were selected because they have been exposed to the clinical environment for a longer period.

### Data collection method

Data collection was done by means of focus group discussions. Five focus group discussions were held, which comprised 6–8 participants each. The interviews were stopped only when data saturation was reached. Focus group discussion has the advantage of collecting viewpoints from many individuals within a short time (Polit & Beck 2013). Similarly, in this study the focus group discussion assisted participants who were initially not free to share their experiences to relax and feel free to express their thoughts, feelings and behaviours. All the focus group discussions were conducted in English. The central question was ‘what are your experiences as a male student in a profession traditionally perceived as a female domain?’; follow-up questions were guided by the responses of the participants. The researcher was assisted by another qualitative research expert during data collection. The first focus group session was conducted by two facilitators so that both facilitators followed the same protocol when conducting subsequent group discussions. The remaining four group discussions were divided between the two facilitators. The interviews were conducted when students had finished with their classes and were relaxed and less likely to be disrupted in a quiet environment at the selected public college of nursing. Each focus group discussion lasted for 45 min.

### Data analysis

Data were transcribed verbatim. A Tesch’s step, as described by Creswell (2011), was used, reading through the transcripts, sorting and organising, and identifying themes and subthemes. The researcher also asked an independent coder, who was familiar with the Tesch’s open code method of data analysis, to read through the data and confirm the identified themes and subthemes.

### Ethical considerations

Permission to conduct the study was obtained from Limpopo Province research ethics committee (Reference: 4/2/2) and Limpopo College of Nursing. Participation in the study was voluntary. The researcher made an appointment with the participants whilst they were at the college for theoretical instruction, to discuss participation in the study. The participant information session was held 2 weeks before the interview, with the aim of clarifying any questions the participants may have had and to build up a rapport with them. This was very important when conducting focus group discussions as it enhanced active participation. During this session the researcher informed the participants about the purpose and significance of the study, that participation is voluntary and about their rights to participate or not to participate. Participants were also made aware that they would not be prejudiced if they chose not to participate or withdraw. Informed consent forms were distributed to participants. Only those who signed the consent took part in the focus group discussion. Permission to record the interviews was sought from the participants prior to commencement of the interviews. Confidentiality, privacy and anonymity were maintained by using codes instead of names.

## Results

During the focus group discussions, themes and subthemes emerged which described the male students’ experiences in a profession perceived to be a female domain at a selected public college of nursing in Limpopo, South Africa (see Table 1).

**TABLE 1:** Themes and subthemes on the experiences of male student nurses in a 4-year nursing programme in Limpopo College of Nursing.

Themes	Subthemes
1. Discrimination in the clinical setting	1.1 Delegation of non-nursing duties 1.2 Not given preference for learning opportunities
2. Lack of role modelling and mentoring	2.1 Feeling lost or not belonging 2.2 Recruitment bias leading to isolation 2.3 Use of feminine terms

### Theme one: Discrimination in the clinical setting

Participants felt that they were not treated the same as their female counterparts in the nursing programme. The participants felt that they were being discriminated against because of their gender in the clinical setting. The following subthemes were identified: (1) delegation of non-nursing duties and (2) not given preference for learning opportunities

#### Subtheme one: Delegation of non-nursing duties

The participants reported that they were delegated non-nursing duties which were viewed as ‘manly’ whilst their female counterparts were delegated nursing duties. The above is depicted by the following statements:

‘We [male student nurses] are allocated to clean trolleys, we are basically delegated to assist the general workers to do their job.’ (FDG 1, P1)

The above statement was confirmed by another participant who put it like this after taking a deep breath and looking a bit tensed:

‘Just as you come through the door you are told to push a bed, take the patient to the X-ray department and when coming back you are again told that there are things that need to be taken to another department.’ (FDG 2, P2)

‘[*J*]ust like the day a high-ranking official was visiting one of the hospitals, we [*male student nurses*] were allocated to clean trolleys, wash windows, were basically delegated to assist the general workers to do their job, and when we were complaining to say these are non-nursing duties, we got remarks like ‘other males are working in mines.’ (FDG 4, P 3)

These statements raise a concern regarding the quality of the end products when male students lose an opportunity to practise nursing skills. The participants in the study felt disgruntled with the non-nursing duties that they were expected to carry out. These nurses are all trained to render quality nursing care; thus they all should be afforded an opportunity to practise nursing skills. It is important to ensure that the discrimination in terms of delegation in the clinical environment and exposure of students to available learning opportunities are to be prioritised.

#### Subtheme two: Not given preference for learning opportunities

The participants felt that they lost opportunities to practise nursing skills. They also felt that they are all trained to render quality nursing care. Learning opportunities are important for learners to gain the experience in the clinical environment. However, findings revealed that participants were called when there was a need for manpower whilst their learning needs were unmet. Failure to be given the opportunity to learn was indicated as follows:

‘The other issue is that of “man power”, each time there is a non-nursing duty that need to be done, they say “Where is a male student?”, but when there are better things like insulin checking workshop they decide to take female students. So, we are not prioritised for things relevant to our learning as student nurses. We are seen as people who can be used to lift up heavy things, which is not part of our scope or learning.’ (FDG 3, P 1)

Another student supported the above statement as follows:

‘In most cases female nurses prefer to work with other females, even when, for example, I am delegated to give injection. When I want to prepare the trolley for injection a female nurse would say “Do not worry I will prepare and call you when we have to give the injections”, when time comes she would just call a female student nurse instead of me.’ (FDG 4, P 1)

Male nursing students felt uncertain of their training as they were in most cases not exposed to workshops and relevant clinical skills. It is important that the male nursing students are exposed to clinical learning that will improve their knowledge so that they can render quality patient care. In addition, the inadequate exposure of male student nurses to clinical skills can compromise the quality of nursing care rendered to patients.

### Theme two: Lack of role models and mentoring

Participants felt that they lacked role models; three subthemes emerged as (1) feeling lost and not belonging, (2) recruitment bias which led to isolation and (3) use of feminine terms.

#### Subtheme one: Feeling lost and not belonging

Findings of the study revealed that participants experienced feelings of being misfits and actually felt lost. These feelings were reinforced by the behaviour of female colleagues, such as gossiping which male students felt was unacceptable. Furthermore, participants expressed that they found nursing units to be a female-dominated environment where they also experienced unwelcoming remarks from female nurses, who were their seniors in the clinical settings. This was evidenced by the following quotes:

‘We got remarks like other males are working in mines.’ (FDG 3, P 3)

‘When the ward is not busy, I would just be sitting alone and cannot go out because I’m on duty but I feel I do not belong.’ (FDG 2, P 6)

‘In the clinical setting, the female professional nurses in most cases enjoy dictatorship, they do not want any reasoning or being questioned.’ (FDG 4, P 3)

The participants indicated that in most cases especially in units where there were more female nurses, they would feel like they do not belong because they would not be able to engage in a conversation with their colleagues or seniors. The findings of the study also suggested that the female nurses in the wards were autocratic and did not take to being questioned politely and did not want people who want to reason with them. The remarks from senior staff members were discriminatory and perpetuated the idea that nursing was meant for women only which is actually not the case.

#### Subtheme two: Recruitment bias which led to isolation

The participants voiced that they felt isolated from other male colleagues because of the fact that each group predominantly comprises women. This was expressed as follows:

‘[*T*]he intake of students in the college is also unfairly done, because there are always more females than males. There should be more males who are encouraged to do nursing. It is important that during career guidance people be told about the goodness of nursing, so that they can be attracted to nursing. The career exhibition should include young and vibrant male nurses of which students of both sex at high school can associate with.’ (FDG 3, P1)

‘More males to be recruited in to the profession so as to close the gap in terms of the number of females being more compared to males and community awareness about what nursing is all about, so that they can appreciate the fact that nursing is not only for females.’ (FDG 3, P 3)

‘Since from first year I find that I’m allocated in the ward being the only male student with four other [*sic*] female students and in other wards there is no male nurse at all. So I would find myself being the only male. Wards like medical, surgical and paediatric wards are more female-dominant wards and females like to gossip, so as a male you are isolated with no one to talk to.’ (FDG 5, P 2)

‘Males prefer to work in busy departments like the casualty, and occupational health clinics, infection control units and psychiatric units where there are more males to interact with, so most of the time I found myself being the only male person among females.’ (FDG 1, P 4)

These findings demonstrate that although males have a passion for patient care, female-dominated environments overwhelm them. Thus, they prefer to work in units where there are more men to associate with. However, this leads to the isolation of male students, which is further perpetuated by the imbalances when recruiting students for the programme where male students are always in the minority compared to female students. The participants in this study expressed their views of the need to balance the number during student intake.

#### Subtheme three: The use of feminine terms

Findings of the study revealed that participants viewed that terms used in the profession such as ‘sister or nurse’ when referring to professional nurses as unacceptable. In addition, participants felt that these terms describe the nursing profession as inherently female oriented. This was evidenced by the following statements:

‘They’ll have to change the term nurse, because it is female related, traditionally we know nursing to belong to women.’ (FDG 3, P 2)

‘I also concur, the term sister applied to professional nurses is also embarrassing because a sister is a female person, immediately you tell people you are doing nursing you get comments like “oh so you are going to be a sister?” How can I be a sister when I am a male person?’ (FDG2, P5)

‘[*T*]he term ‘sister’ does not go well with me too, to a point that if a patient calls me a doctor, truly speaking I do not correct the patient and I find it hard to respond when a patient is calling me “sister”. It usually feels like I am accepting a female role.’ (FDG2, P3)

‘One male professional nurse once told us that he did not wear his distinguishing devices because he did not want to be called a sister.’ (FDG 3, P4)

The participants in this study indicated that the use of feminine terms such as ‘sister’ and ‘matron’ was distasteful to them and that it also perpetuated the perception that nursing was meant for women. They further indicated that there was a need to discontinue the use of such terminology. It was ironic that patients would mistakenly call the male nursing students doctors. The question is was this because being a doctor is viewed as more suitable for a male person or was it because patients found it hard to call a male person ‘nurse or sister’?

## Discussion of findings

### Outline of results

Findings revealed that participants were experiencing discrimination in the clinical setting, where they were delegated non-nursing duties and that their learning was not prioritised. They further indicated that their female colleagues were afforded more opportunities in practising nursing tasks, whilst they were delegated perceived ‘manly’ tasks such as lifting heavy objects or patients or pushing patients to other departments like X-ray. Rajacich et al. (2013) and Achora (2016) support the study findings by affirming that male nurses in their study were often delegated non-nursing duties because they were perceived as being able to do the heavy work which female nurses perceived needed ‘man’ power. Results revealed that some participants were delegated to clean trolleys and wash windows, which imply that male nurses are often utilised to perform non-nursing duties.

In South Africa, the constitution does not discriminate. In Chapter 2 section 9, it states that no one should be discriminated against in terms of gender (Republic of South Africa 1996). The discrimination may significantly impact on male students’ learning and the quality of patient care. This discrimination may also explain why most male nurses do not prefer working in general clinical units but rather in specialised units, where there are usually more male nurses.

The findings of the study suggest that female student nurses were given more opportunities to attend workshops and seminars related to nursing skills whilst male student nurses were overlooked when such opportunities were available. This is an indication that men in the clinical environment are overlooked and women are given preference and this brings dissatisfaction among men. Participants in this study indicated that it seemed they were only noticed when difficult tasks were to be carried out even if it was not part of their learning requirement. In addition, participants further reported that even when they were delegated nursing duties, senior staff members would still call a female student to do the task.

In support of the findings of this study, in a study conducted by Wang et al. (2010) in China on the perception of nursing and learning experiences of male student nurses, participants also reported being discriminated against their female colleagues. Ross (2017) also came to similar findings where participants affirmed that nursing instructors tended to discriminate against male student nurses when teaching nursing skills and concentrated more on female than male students.

Findings of the study revealed that participants felt lost in female-dominated clinical units and that they missed certain male traits during their allocation in these wards. Buthelezi et al. (2015) support the study findings by indicating similar findings about feelings of being lost and not belonging. These feelings of not belonging are related to few male role models in clinical settings (Stanley 2012). According to Mott and Lee (2017) in a study on ‘Navigating unfamiliar waters: Men in nursing academia’, male student nurses do not have opportunities to discuss their concerns and anxieties with other men or to see men in nursing practice. Bartfay et al. (2010) supported the argument that men because of their minority status in nursing experience ‘role strain’, which they defined as feeling unwelcomed. In the selected clinical environment, clinical placement is often done in groups, which have single or no male members. The implication is that male student nurses are often exposed to female-dominated environments leaving them with a feeling of not belonging.

The feeling of not belonging was at times worsened by the unwelcoming remarks towards participants from some professional nurses, which suggest that nursing is for women. The findings about male students being not welcomed by some female professional nurses in this study concurs with what was reported by Kirk, O’Lynn and Ponton (2013) where participants experienced similar discriminatory remarks or were told by some instructors that they were invading women’s territory in nursing or that nursing was not a profession for men.

Christensen and Knight (2014) in ‘a thematic analysis of the experiences of male student nurses’ also reported discrimination against male student nurses. Findings of the study further revealed that some senior female professional nurses were acting as dictators and not accepting advice or being reasoned with. The implication is that participants were to take whatever they were told without reasoning or questioning. In support of these findings, Mott and Lee (2017) assert that the difference in ways of communication and thinking between men and women often creates tension.

The findings of the study revealed that the recruitment strategy used in the college seems to favour women, thus creating an imbalance in the number of male and female students. This imbalance means that in some instances a male nurse may find himself being the only male person in the ward. Participants suggested that in order to attract more men to the profession, male nurses should be part of the marketing team. Sayman as cited by Meadus and Twomey (2016) highlighted the need to market nursing in a more androgynous manner so as to attract more men in to the nursing profession. The implication is that the presence of more men in the profession would probably also change the stereotyping of nursing as a female profession. According to the student affairs enrolment register (2013–2017), the ratio of men to women for a period of 5 years was 1:3. This implies that more women have been enrolled into the programme perpetuating the perception that nursing is a female domain.

The findings of the study revealed that feminine terms including ‘nurse’, ‘nursing’ and ‘sister’ perpetuated the female stereotype. These feminine terms were causing title confusion and were frustrating to participants as at times frustrating to participants as they were mocked by people saying so when you qualify you will be a ‘sister’ and it sounded as if their gender would change. The implication is that the terms commonly used in the nursing profession are not readily welcomed by male nurses. Some participants indicated that they were at times mistakenly called doctor instead of nurse by patients and would not correct them because that was better than being called a sister. This is congruent with findings by Achora (2016), where participants also said they were often mistaken for doctors by patients. Wolfenden (2011) describe what he calls the ideal Victorian family with the male being a doctor and head of the family the woman is a nurse. This suggests that there are expectations placed for each gender on what is viewed as appropriate or inappropriate. Concerns about the use of feminine terms in the nursing profession were echoed by Boucher (2011) and Kulakac et al. (2009) where participants also reported that the use of the terms ‘sister, nurse, nursing’ was feminine and does not accommodate male nurses. The findings of the study suggested a need to change these feminine terms so as to be more welcoming to the male nurses in the profession.

### Measures to ensure trustworthiness

To enhance trustworthiness, the following criteria were adhered to: credibility, transferability, dependability and conformability as according to reference. To ensure credibility, the researcher discussed the themes and subthemes that developed with the participants in order confirm that the data were correctly captured. Peer debriefing was also done with expert peers, internally during the data collection process. At the end of the study, an external reviewer was engaged to confirm the coding of the researcher. Dependability and conformability were enhanced by an inquiry audit, in which the documents were scrutinised by an experienced independent coder. The researcher developed an audit trail consisting of audio tapes, field notes and transcripts. Transferability – the researcher gave a thick description of the setting, process of data collection, analysis methods, findings and literature control to enhance comparison by other researchers who may be interested in studying a similar topic.

### Limitations

The study was conducted in one specific public college of nursing in Limpopo; thus, the results may not be generalised as the experiences in other settings may be different. The sample included only third- and fourth-year students enrolled for a 4-year diploma programme at a selected public college of nursing in Limpopo, South Africa. Therefore, the findings cannot be generalised to all male students who may be enrolled for other programmes such as critical care nursing, emergency nursing, 1-year diploma in psychiatric nursing, diploma in general nursing (bridging), 1-year diploma in midwifery and others.

## Conclusion

The existing gender stereotyping in the nursing profession needs to be addressed, changing the perceptions that nursing is a female profession. In South Africa, attracting more men to the nursing profession may alleviate the on going shortage of nurses and also assist in ensuring that there is even distribution of male and female nurses in the clinical units so that male students may have mentors. Men who choose nursing make a choice because of their passion to care for the ill and need to be assisted to fulfil their dreams. A greater number of male nurses will probably influence a change in the mind set of other nursing staff members who still perceive nursing as a female profession. This study recommends that male nurses should be trained comprehensively, so that they are able to give nursing care in a variety of settings. Furthermore, clinical placement should be fully utilised to benefit all students regardless of their gender, and recruitment of more men into the nursing profession should be prioritised.
